# The protective effects of antioxidants against endogenous and exogenous oxidative stress on bull sperm

**DOI:** 10.1007/s11626-024-00944-w

**Published:** 2024-07-30

**Authors:** Ali MD Younus, Takahiro Yamanaka, Masayuki Shimada

**Affiliations:** 1 Graduate School of Integrated Sciences for Life, Hiroshima University, Higashihiroshima, Hiroshima 7398528 Japan; 2 Graduate School of Innovation and Practice for Smart Society, Hiroshima University , Higashihiroshima, Hiroshima 7398528 Japan

**Keywords:** Mitochondria, Reactive oxygen species (ROS), Antioxidants, Frozen-thawed sperm, Sperm motility

## Abstract

Oxidative stress, caused by both endogenous and exogenous factors, affects sperm function by damaging morphology and reducing metabolic activity, leading to reduced fertilization ability. The purpose of this study was to investigate the effects of oxidative stress on bull sperm and to evaluate the efficacy of targeted antioxidants in mitigating these detrimental effects. Fresh bull semen samples were subjected to hydrogen peroxide (H_2_O_2_) and antimycin treatments to induce oxidative stress, and the antioxidants PQQ, ergothioneine, and vitamin C were applied to counteract the induced stress. Sperm motility, viability, and reactive oxygen species (ROS) levels in the cytoplasm and mitochondria of sperm were assessed using computer-assisted sperm analysis (CASA) and flow cytometry. The treatment with H_2_O_2_ rapidly decreased sperm viability, and antimycin-induced mitochondrial ROS mainly decreased sperm motility; PQQ and vitamin C effectively reduced mitochondrial ROS, while ergothioneine and vitamin C reduced cytosolic ROS. In frozen-thawed sperm, oxidative stress was elevated in both cytoplasm and mitochondria, and all three antioxidants improved sperm motility by inhibiting ROS production. Furthermore, the localization of oxidized lipids (4-hydroxynonenal) in sperm was detected using immunofluorescence, indicating that oxidative stress affects the head and midpiece of sperm. These findings highlight the potential of targeted antioxidants to mitigate the detrimental effects of oxidative stress on bull sperm and provide valuable insights to improve semen quality and optimize the use of antioxidants in artificial insemination.

**Supplementary Information:**

The online version contains supplementary material available at 10.1007/s11626-024-00944-w.

## Introduction

Artificial insemination in bovine is an important tool for improving reproductive efficiency, and using high-quality semen directly contributes to the productivity and economics of the livestock industry. One of the known factors in the decline of sperm quality is oxidative stress (Simões *et al.* 2013; Aitken 2020). The oxidative stress not only damages important components of the spermatozoa, such as the axon part of the tail, acrosome, and midpiece, but also negatively affects sperm function by impairing the metabolic activity involved in ATP production in both the mitochondria and cytoplasm (Aitken *et al.* 1998; Moazamian *et al.* 2015). Oxidative stress triggers apoptosis in the spermatozoa, leading to DNA fragmentation (Lewis and Aitken 2005; Aitken and Baker 2006; Bansal and Bilaspuri 2011; Agarwal *et al.* 2014b). Essentially, oxidative stress not only diminishes the sperm's capacity to fertilize oocytes both *in vitro* and *in vivo*, but also impedes zygote development caused due to chromosomal abnormalities.

Reactive oxygen species (ROS) generated by both endogenous and exogenous factors oxidize various biomolecules, including lipids, proteins, and nucleic acids. These oxidative processes compromise the functionality and stability of these biomolecules and are collectively termed as oxidative stress (Morielli and O’Flaherty 2015; Panner Selvam *et al.* 2020). Endogenously, ROS are byproducts of ATP synthesis in the mitochondrial electron transport chain. Normally, ROS are neutralized by the glutathione reduction pathway. However, under conditions of rapid ATP production, ROS generation surpasses the capacity for reduction, thereby exacerbating oxidative stress within the mitochondria and cytoplasm. In addition, mitochondrial damage caused by excessive calcium influx disrupts the electron transport chain resulting in ROS production (Aitken *et al.* 2016). Since sperm motility depends on ATP synthesis, dysfunction of mitochondria, which are the ATP synthesis organs, significantly impairs sperm motility (Bulkeley *et al.* 2021). Although addition of substrates, such as glucose and fatty acids, temporarily boosts motility, sustained ATP overproduction exacerbates oxidative stress, ultimately diminishing sperm motility (Zhu *et al.* 2019; Islam *et al.* 2021).

Extracellular factors can also induce oxidative stress in sperm. Leukocyte-derived ROS, produced in response to bacterial infections in semen, eradicate bacteria, however, inadvertently damage the sperm (Agarwal and Said 2005; Agarwal *et al.* 2014a; Fraczek *et al.* 2016). Cryopreservation-induced fluctuations in osmotic pressure and temperature also generate ROS both inside and outside the cells, altering sperm lipids and protein molecules (John Morris *et al.* 2012; Pini *et al.* 2018). Moreover, organelle damage during freezing and thawing releases ROS into the cytoplasm, promoting lipid peroxidation and impairing sperm motility, viability, and function (Chatterjee *et al.* 2001; Chatterjee and Gagnon 2001).

Various antioxidants naturally present in the seminal plasma mitigate oxidative stress, constituting primary defense against free radical-induced damage (Lewis *et al.* 1997; Bansal and Bilaspuri 2011; Sharma *et al.* 2013). However, in frozen-thawed semen, antioxidant activity may be lesser due to semen dilution or the removal of seminal plasma. Consequently, addition of exogenous antioxidants is necessary to mitigate oxidative stress. Small-molecule antioxidants include vitamin C, vitamin E, glutathione, ubiquinol, coenzyme Q10, PQQ, and peroxiredoxins (PRDXs), whereas large-molecule antioxidants include superoxide dismutase (SOD), catalase, glutathione peroxidase (GPx), and albumin. These antioxidants exhibit varying mechanisms of action, necessitating investigations into the factors that effectively counteract specific oxidative stress to ensure sperm normality (O’Flaherty and Scarlata 2022). Therefore, the aim of this study was to elucidate the antioxidant efficacy of fresh bull semen samples treated with hydrogen peroxide and antimycin against endogenous and exogenous oxidative stress. Additionally, the potential applicability of these findings in improving the viability of freeze-thawed bull semen will be explored.

## Materials and Methods

### Semen collection and incubation of sperm

The Livestock Improvement Association of Japan, INC (Tokyo, Japan) provided fresh semen from Japanese breeding bulls that was collected using an artificial vagina and diluted with TRIS-egg yolk extender. The fresh semen was placed in thermos bottles and transported to the laboratory within 4 h at 4°C. The semen sample was washed twice through centrifugation (400 × g for 3 min) using the modified Human Tubal Fluid (mHTF) medium (Umehara *et al.* 2020). After centrifugation, the sperm pellet was resuspended in mHTF medium containing pyrroloquinoline quinone (PQQ) disodium salt (BioPQQ^®^; Mitsubishi Gas Chemical Co., Inc., Tokyo, Japan), ergothioneine (14905, Cayman, Ann Arbor, MI) or vitamin C (A5960, Sigma–Aldrich, St Louis, MO), and was used for all analyses. Some samples were treated with H_2_O_2_ (20779-65, Nacalai Tesque, Osaka, Japan) or antimycin (A8674; Sigma) to examine the effects of ROS induction. The samples were incubated at 37°C in a humidified atmosphere containing 5% CO_2_.

The frozen bull semen was a gift from the Livestock Improvement Association of Japan, Inc. The 0.5 ml straw of frozen semen was thawed in water at 37°C for 30 s and immediately diluted with 6 ml of mHTF medium. In the first thawing medium, 1 µM PQQ, 100 µM ergothioneine or 1000 µM vitamin C was added to the mHTF medium. The first thawing medium containing frozen-thawed sperm was centrifuged at 300 *g* (5 min at 37°C), and the sperm pellet was washed twice with each medium. After centrifugation, the sperm pellet was resuspended in each medium and used for all analyses. The samples were incubated at 37°C in a humidified atmosphere containing 5% CO_2_.

Table 1 lists information on the bulls used in the experiment. Sperm motility was assessed before every experiment, and all experiments were performed when a percentage of motile showed motility over 60%.

**Table 1.** Semen and bull biological characteristics

ID	Semen volume (mL)	S.D.	Age (year)
No.1	7.26	± 1.71	5
No.2	7.45	± 1.60	5
No.3	5.67	± 1.60	5
No.4	4.73	± 1.22	4
No.5	6.36	± 1.08	9
No.6	5.03	± 1.11	10
No.7	7.21	± 1.38	6

Semen volume was calculated from 12 to 40 ejaculations. A percentage of motile sperm greater than 60% was used in the experiment. S.D.; Standard deviations

### Assessment of sperm motility

Sperm motility was evaluated using computer-assisted sperm analysis (CASA), as described in our previous study (Islam *et al.* 2021). Approximately, 3 µL of sample was placed in a pre-warmed counting chamber for CASA after incubating the sperm at different time intervals. Sperm tracks (0.5 s and 45 frames) were captured at 60 Hz according to our previous study using the CASA system (HT CASA-Ceros II; Hamilton Thorne, Beverly, MA). A minimum of 200 spermatozoa were assessed to carry out each CASA. Single sperm motility was calculated by multiplying motility by the total concentration.

### Determination of ROS by flow cytometry

Photooxidation-resistant dichloro-dihydro-fluorescein diacetate (DCFH-DA) is a total ROS (H_2_O_2_, superoxide anion, and hydroxyl radical) probe (R253; DOJINDO LABOLATORIES Co., Ltd., Kumamoto, Japan). According to the manufacturer’s protocols, sperm was pretreated with a photo-oxidation-resistant DCFH-DA working solution and incubated for 30 min at 37°C in a humidified atmosphere of 5% CO_2_. The sperm was washed twice with mHTF. After removing the supernatant, the sperm was mixed with mHTF-containing antioxidants (PQQ, ergothioneine, or vitamin C) and/or inducers of oxidative stress (H_2_O_2_ or antimycin). After 0, 1, 2, 3 and 4-h of incubation, the samples were analyzed using a flow cytometer (Attune®NxT Acoustic Focusing Cytometer, Thermo Fisher Scientific Inc., Waltham, MA). Green fluorescence (DCF) was evaluated using a 488 nm laser and a 530/30 nm bandwidth filter. Data are expressed as the percentage of fluorescent-positive sperm. Cutoff values were set using an unstained sample. The gating strategy is illustrated in Supplementary Fig. S1. Localization of fluorescence in the sperm was determined using an APX100 Digital Imaging System (EVIDENT Co., Ltd., Tokyo, Japan).

### Mitosox deep red assay

Generation of mitochondrial superoxide anion was investigated according to the MitoSOX™ deep Red Assay Kit (MT-14, DOJINDO LABORATORIES). The sperm was pretreated with MitoSOX™ deep Red working solution, incubated at 37°C for 30 min and washed twice with mHTF. After removing the supernatant, the sperm was mixed with mHTF-containing antioxidants (PQQ, ergothioneine, or vitamin C) and/or inducers of oxidative stress (H_2_O_2_ or antimycin). The samples were analyzed by flow cytometry after 0, 1, 2, 3 and 4-h of incubation. Fluorescence was evaluated using a 638 nm laser and a 570/14 nm bandwidth filter. Data are expressed as the percentage of fluorescent-positive sperm. Cutoff values were set using an unstained sample. The gating strategy is illustrated in supplementary Fig. S1. Localization of fluorescence in the sperm was determined using a Nikon AX confocal microscope (Nikon Solutions Co., Ltd., Tokyo, Japan).

### Mitochondrial activity

Mitochondrial activity of sperm was measured using a MitoPT® JC-1 Assay Kit (911, Immuno Chemistry Technologies, LLC, Bloomington, MN) according to our previous study. Briefly, sperm were incubated with 200 µL of working solution containing 5,5’,6,6’-tetrachloro-1,1’,3,3’-tetraethylbenzimidazolyl carbocyanine iodide (JC-1) dye at 37°C for 30 min in the dark. The sperm suspension was centrifuged and washed twice with mHTF medium. After washing, the sperm pellet was resuspended in mHTF medium and analyzed through flow cytometry using a 488 nm laser and filters with bandwidths of 530/30 and 574/26 nm. Data are expressed as the percentage of fluorescent-positive sperm. The gating strategy is illustrated in supplementary Fig. S1. A total of 50,000 sperm events were analyzed.

### Immunofluorescence

Sperm was mounted on glass slides, air-dried, fixed with 4% paraformaldehyde for 30 min, and permeabilized with 0.3% (v/v) Triton X-100 in PBS for 30 min at room temperature. After washing with PBS, samples were incubated with blocking solution from the MOM Kit (MKB-2213, Vector Laboratories, Newark, CA) at 25℃ for 30 min to block nonspecific sites. The samples were then incubated at 4℃ overnight with primary mouse antibody, anti-4 hydroxynonenal antibody (4HNE; 1:100; ab48506; Abcam, Cambridge, UK). After washing with PBS, the antigens were visualized using Cy3-conjugated sheep anti-mouse IgG (1:100; C2181, Sigma). Digital images were captured using an APX100 Digital Imaging System.

### Statistical analysis

At least three animals were used for the fresh semen study, and the experiments were replicated at least three times in each group. Five animals were used in the frozen semen study. Quantitative data were presented as mean ± SEM. Percentage data were subjected to arcsine transformation before statistical analysis. Motile single-sperm data were analyzed using a paired t-test with p-value correction using the Bonferroni method for multiple tests. Differences between groups were assessed using one-way analysis of variance (ANOVA). When the ANOVA was significant, differences among values were analyzed using Tukey's honest significant difference test for multiple comparisons. Dunnett's test was used to analyze the ROS inducer/antioxidant combination (Fig. 4) and frozen sperm experiments (Fig. 5). The comparison controls are listed in the figure captions below. R (version 4.3.1) was used for statistical analysis. Statistical value of *p*<0.05 was defined as a significant difference.

## Results

### Changes in motility and intracytoplasmic and mitochondrial ROS levels of fresh sperm with incubation time

**Figure 1.**
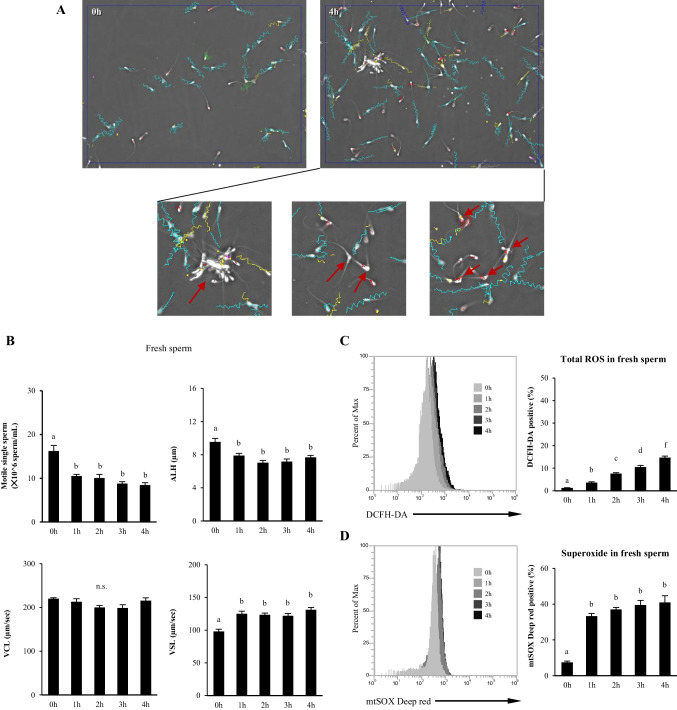
Significant decrease in viability and an increase in ROS levels of fresh bull sperm during the incubation period. (***A***) Tracks of sperm incubated for 0 and 4 h were determined using the CASA system. *Red arrows* indicate the aggregated sperm, which is the cause of overestimating CASA parameters because it lowers the number of dead sperm. (***B***) Changes in fresh sperm motility single counts implying sperm viability and in kinetics patterns with incubation time. (***C*** and ***D***) Time-dependent changes in total ROS in the cytoplasm or mitochondrial superoxide level of fresh bull sperm detected using DCFH-DA or mtSOX deep Red, respectively. Flow cytometric patterns and percentage of positive sperm were shown. Different *letters* represent significantly different groups (*p* < 0.05).

The number of motile single sperm and ALH (µm) significantly decreased from 0 to 4 h of incubation (Fig. 1*A* and *B*). The VCL (µm/sec) did not differ from 0 to 4 h of incubation (Fig. 1*B*). However, in VSL (µm/sec) highly significant differences were found from 0 to 4 h of incubation (Fig. 1*B*). The ROS levels in the cytoplasm and mitochondria of fresh samples were measured using two probes (DCFH-DA and mtSOX deep red). The total ROS levels significantly increased in a time-dependent manner in fresh sperm from 0 to 4 h of incubation (Fig. 1*C*). However, mitochondrial ROS levels increased sharply for up to 1 h of incubation and remained significantly higher for up to 4 h. Thus, both total ROS levels and mitochondrial ROS levels increased in different patterns with incubation, and cell viability greatly reduced on incubation. However, the relationship between them remains unclear.

### Induced oxidative stress in fresh semen using H_2_O_2_ and antimycin

**Figure 2.**
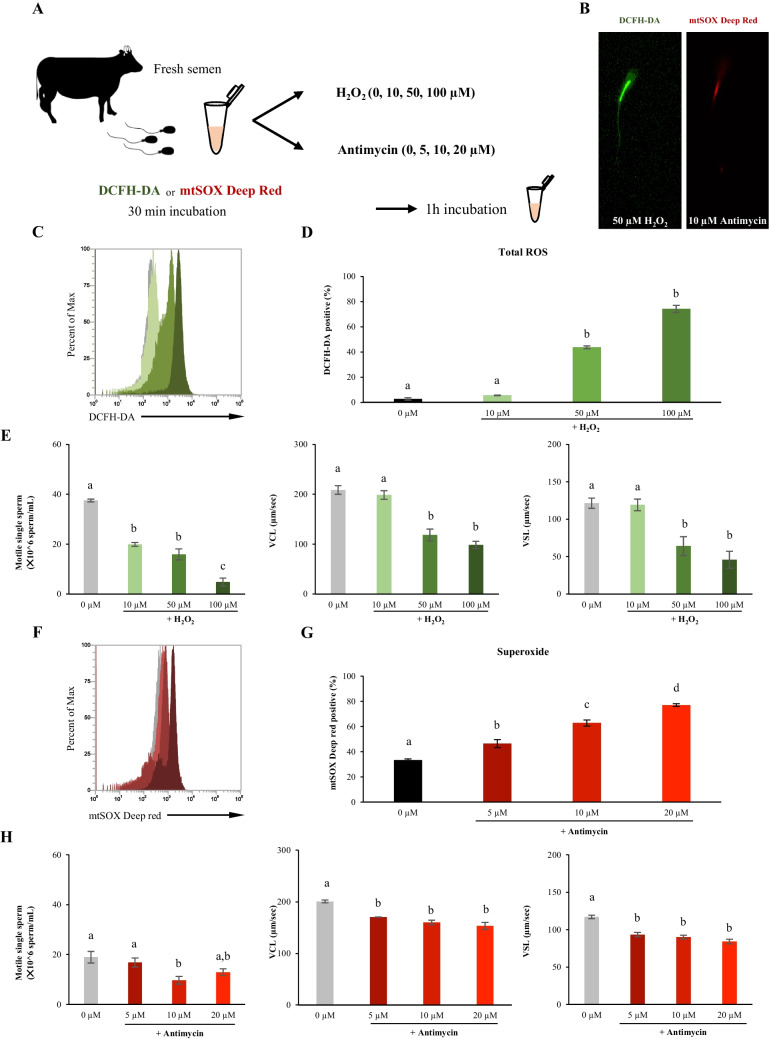
The impact of oxidative stress inducers, H_2_O_2_ and antimycin, on fresh bull sperm motility and ROS levels. (***A***) Experimental design to investigate the effect of the oxidative stress inducers on fresh bull sperm. (***B***) Representative images of the sperm fluorescence signal with DCFH-DA and mtSOX deep Red. *Left*: DCFH-DA fluorescence image of sperm treated with H_2_O_2_. *Right*: mtSOX deep Red fluorescence image of sperm treated with antimycin. (***C***) Flow cytometric patterns of DCFH-DA. (***D***) Percentages of DCFH-DA positive sperm. (***E***) Sperm kinetics changed by H_2_O_2_. (***F***) Flow cytometric patterns of mtSOX deep Red. (***G***) Percentages of mtSOX deep Red positive sperm. (***H***) Sperm kinetics changed by antimycin. Different *letters* represent significantly different groups (*p* < 0.05).

To elucidate the relationship between the three factors that were unknown in the above experiments, fresh bull sperm was treated with two different oxidative stress inducers, hydrogen peroxide or antimycin (Fig. 2*A*). After one hour of incubation, sperm motility and ROS levels were examined using DCFH-DA and mtSOX deep probes. Percent of positive sperm stained using DCFH-DA (total ROS positive) differed significantly from the control group on treatment with more than 50 µM of H_2_O_2_ (Fig. 2*B*-*D*). However, the motile single sperm percentage significantly reduced by treatment with more than 10 μM H_2_O_2_ (Fig. 2*E*). On the contrary, the motion speeds (VSL and VCL) significantly reduced at concentrations of 50 μM or more (Fig. 2*E*). The percentage of mtSOX deep red-positive sperm significantly increased on treatment with antimycin in a dose-dependent manner (Fig. 2*F* and *G*). Although the motile single sperm ratio slightly decreased due to the treatment, the VCL (µm/sec) and VSL (µm/sec) significantly reduced in a dose-dependent manner (Fig. 2*H*). Thus, the viability of sperm was sensitively reduced by treatment with hydrogen peroxide, whereas the motility rate of the sperm was reduced by an increase in endogenous ROS in the mitochondria of fresh bull sperm.

### Effect of different antioxidants on total ROS and mitochondrial ROS levels under the presence of different oxidative stress inducers

**Figure 3.**
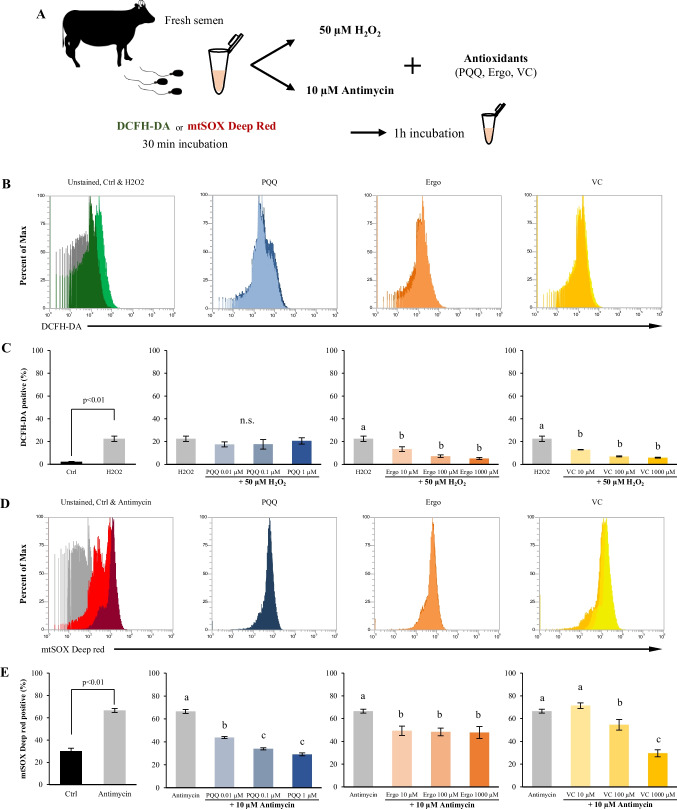
Impact of antioxidants on total ROS and mitochondrial ROS levels in fresh bull sperm under oxidative stress inducers. (***A***) Experimental design to investigate the effect of three antioxidants on induced reactive oxygen species in fresh bull sperm. (***B***) Flow cytometric patterns of H_2_O_2_ and antioxidant-treated sperm stained with DCFH-DA. (***C***) Percentages of DCFH-DA positive sperm. (***D***) Flow cytometric patterns of antimycin and antioxidant-treated sperm stained with mtSOX deep Red. (***E***) Percentages of mtSOX deep Red positive sperm. Different *letters* represent significantly different groups (*p* < 0.05).

The effects of the antioxidant factors, PQQ, ergothioneine, and vitamin C, on extracellular oxidative stress stimulation (H_2_O_2_) and mitochondrial oxidative stress were investigated in fresh bull sperm (Fig. 3*A*). The percentage of sperm showing an increase in total ROS after H_2_O_2_ treatment did not change significantly with PQQ treatment, however a significant dose-dependent decrease was observed in the presence of ergothioneine and vitamin C (Fig 3*B* and *C*). PQQ and vitamin C reduced the percentage of mtSOX deep red-positive sperms (ROS generated in mitochondria by antimycin treatment) in a dose-dependent manner (Fig. 3*D* and *E*). In contrast, there was no dose-dependent change in the ergothioneine levels (Fig. 3*D* and *E*).

### Effect of different antioxidants on sperm motility and ROS level under the presence of different oxidative stress inducers

**Fig. 4.**
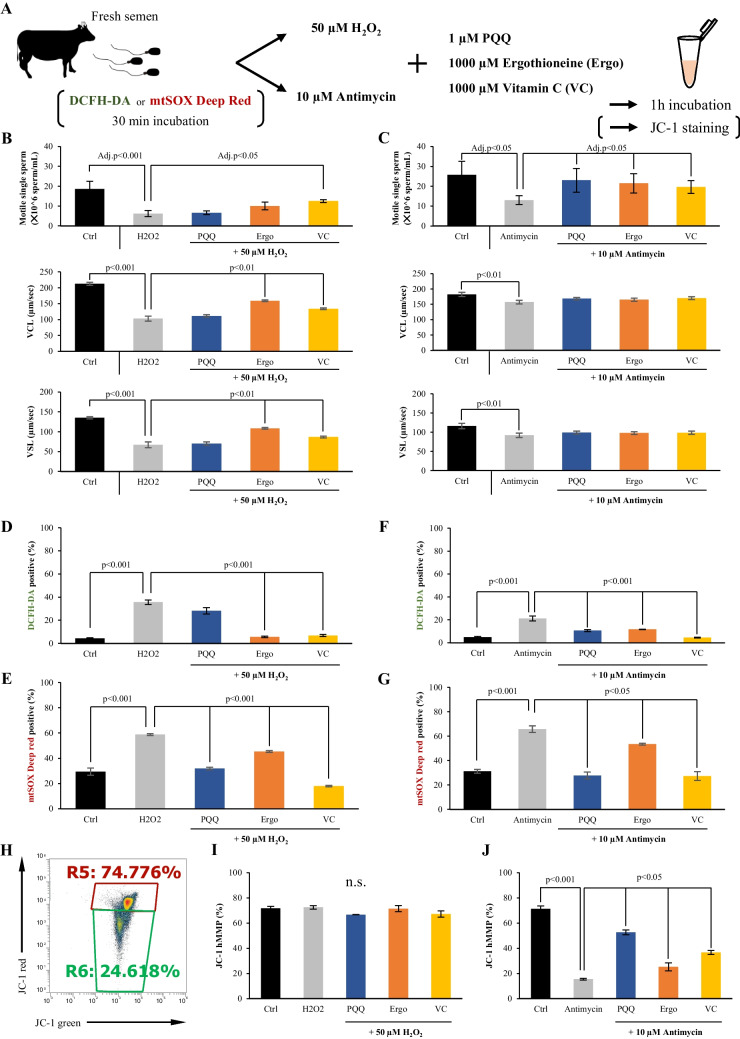
Impact of various antioxidants on fresh bull sperm motility and reactive oxygen species (ROS) levels when exposed to different oxidative stress triggers. (***A***) Experimental design to examine the impact of various antioxidants on sperm motility and levels of reactive oxygen species (ROS) under the two oxidative stress inducers. (***B*** and ***C***) Changes in sperm viability and motility under H_2_O_2_ (***B***) or antimycin (C). (***D***-***G***) Effect of antioxidants on the percentages of DCFH-DA and mtSOX deep Red positive sperm under H_2_O_2_ or antimycin. (***H***-***J***) Measurement of mitochondrial activity using the JC-1 kit. (***H***) Representative flow cytometric pattern of sperm that was not treated with any drug (control) stained with JC-1. Effect of antioxidants on the percentages of high mitochondrial membrane potential (hMMP) under the treatment of H_2_O_2_ (***I***) and antimycin (***J***). H_2_O_2_ or antimycin groups were used as controls for statistical analysis (*p* < 0.05).

The negative effect of H_2_O_2_ on sperm motility was not improved by addition of PQQ or ergothioneine, however a significant improvement was observed only with vitamin C (Fig. 4*B*). Ergothioneines, like vitamin C, significantly increased the motility rate of sperm, which was suppressed by H_2_O_2_ (Fig. 4*B*). In contrast, the negative effect of antimycin on sperm motility was ameliorated by antioxidant factors (Fig. 4*B*). However, none of the antioxidant factors affected the effect of antimycin on the sperm exercise speed (Fig. 4*C*). H_2_O_2_ treatment not only increased the percentage of total ROS-positive sperm, but also increased the percentage of mitochondrial ROS-positive sperm (Fig. 4*D* and *E*). Ergothioneine and vitamin C significantly reduced the percentage of total ROS-positive cells, whereas the percentage of mitochondrial ROS-positive sperm significantly reduced in the presence of all antioxidant factors (Fig. 4*D* and *E*). The effect of antimycin significantly increased not only the percentage of ROS-positive sperm in the mitochondria but also the percentage of total ROS-positive sperm, and the total ROS-positive sperm significantly reduced in the presence of all antioxidant factors (Fig. 4F and *G*). In measuring mitochondrial membrane potential using JC-1, H_2_O_2_ did not affect the high mitochondrial membrane potential (hMMP; Fig. 4*H* and *I*). On the other hand, antimycin significantly reduced the percentage of spermatozoa with hMMPs, but they were rescued by all antioxidant factors (Fig. 4*J*).

### Effect of antioxidants on frozen-thawed bull sperm motility and ROS levels

**Figure 5.**
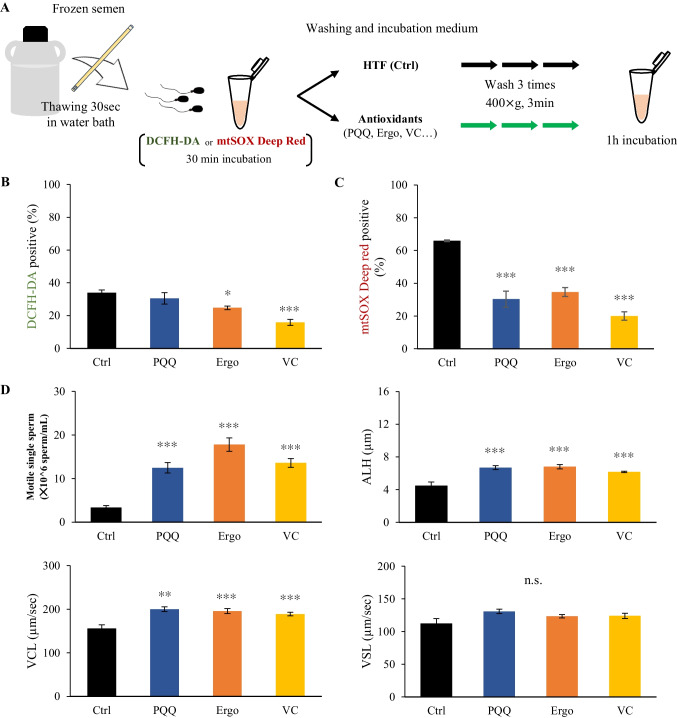
The effect of various antioxidants on frozen-thawed bull sperm motility and ROS levels. (***A***) Experimental design to examine the impact of PQQ, vitamin C, and ergothioneine on frozen-thawed bull sperm motility and ROS levels. Various antioxidants were included in all thawing, washing, and incubation processes using HTF medium as the basic medium. (***B*** and ***C***) Effect of antioxidants on the percentages of DCFH-DA (***B***) and mtSOX deep Red (***C***) positive sperm. (***D***) Changes in frozen-thawed sperm viability and kinetics patterns with antioxidants. *Asterisks* represent significant differences from the control (Ctrl; *: *p* < 0.05, **: *p* < 0.01, ***: *p* < 0.001).

The effects of PQQ, which reduced ROS generation in mitochondria; vitamin C, which reduced both total ROS and ROS in mitochondria; and ergothioneine, which reduced the total level of ROS generated by H_2_O_2_ in frozen-thawed bull sperm were examined (Fig. 5*A*). The percentage of total ROS-positive sperm significantly reduced in the presence of vitamin C and ergothioneine, however not in the presence of PQQ (Fig. 5*B*). However, mitochondrial ROS-positive sperm significantly reduced in the presence of antioxidant factors (Fig. 5*C*). Thus, when frozen-thawed sperm was incubated, oxidative stress elevated in both the cytoplasm and mitochondria. When induction was suppressed by PQQ, vitamin C, or ergothioneine, sperm motility significantly increased in the presence of all antioxidants (Fig. 5*D*). In addition, a significant increase in total mobility speed (VCL) was observed, suggesting that the increase in reactive oxygen species due to both mitochondrial abnormalities and the thawing process in frozen-thawed sperm caused a decrease in sperm motility and mobility speed (Fig. 5*D*).

### Localization of oxidized lipids and the effects of antioxidant factors in fresh or frozen-thawed bull sperm

**Figure 6.**
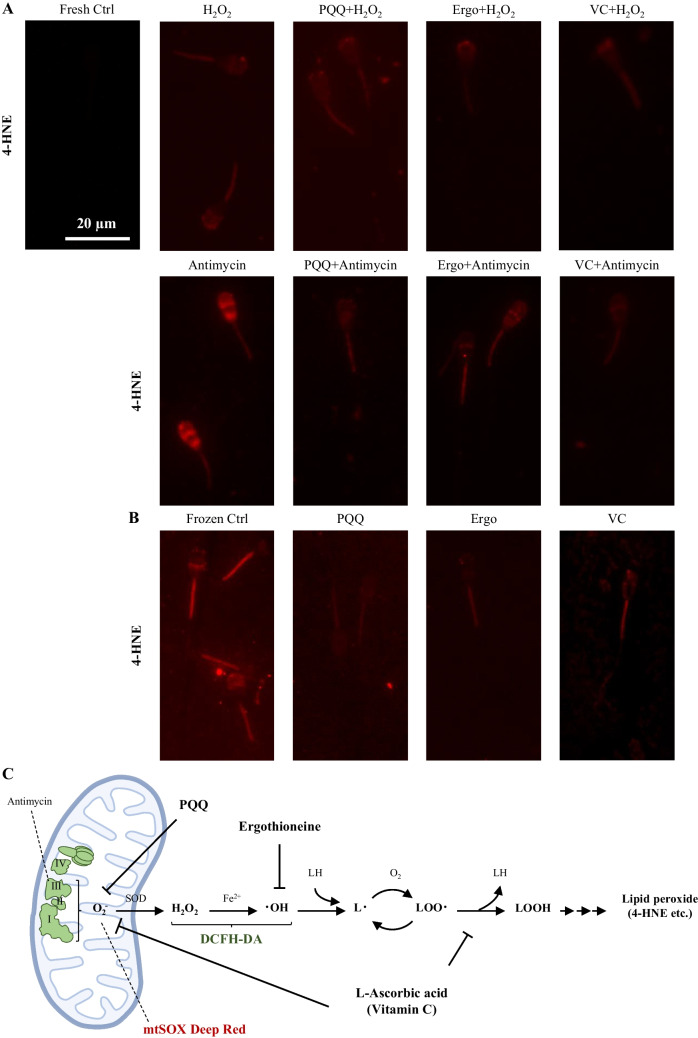
Localization of oxidized lipids in fresh or frozen-thawed bull sperm. (*A*, *B*) Representative images of the sperm fluorescence signal with 4-HNE. (***A***) The ROS inducer/antioxidant combination experiment in fresh sperm. (***B***) The effect of various antioxidants on frozen-thawed sperm. The *scale bar* represents 20 μm. (***C***) The graphical image shows the ROS production cascade in sperm beginning at the mitochondria. It also shows the site of action of the antioxidants used in this experiment.

Localization of oxidized lipids was detected by immunostaining with anti-4-HNE antibodies. Positive 4-HNE signals were strongly detected not only in the middle piece after antimycin treatment but also in the sperm head. These positive signals were attenuated by antioxidant factors. Similarly, positive 4-HNE signals were detected in the sperm head and midpiece following H_2_O_2_ treatment. The 4-HNE positive signals were not abated by PQQ, however were suppressed by ergothioneine and vitamin C (Fig. 6*A*). These results show the same trend as the ROS-positive sperm detection results. Furthermore, from the results of the experiments with frozen-thawed sperm, strong signals were detected in the midpiece, which reduced in the presence of PQQ and vitamin C, and these were the same as in the detection experiment of ROS-positive sperm (Fig. 6*B*).

## Discussion

The results obtained from this study suggest that sperm function was inhibited due to oxidative stress in both the cytoplasm and mitochondria (Fig. 6*C*). In particular, it was thought that viability declined sharply due to oxidative stress caused by H_2_O_2_ from outside the cell, rather than from the mitochondria. In an experiment investigating lipid oxidation, strong signals were detected in the sperm head region after H_2_O_2_ treatment, consistent with a previous report showing that the normality of the acrosome cap is linked to sperm survival (Nagy *et al.* 2004; Fayyaz *et al.* 2017; Guidobaldi *et al.* 2017; Islam *et al.* 2023). In contrast, elevated ROS levels in the mitochondria reduced sperm viability. Mitochondrial dysfunction may lead to the release of large amounts of cytochrome c and calcium within the mitochondria, which may adversely affect sperm survival. In addition, oxidative stress in the mitochondria reduces sperm motility, even at levels that do not affect sperm viability. It has been reported that decreased sperm mobility was due to the oxidation of mitochondrial DNA, mitochondrial transcription factors, and mitochondrial RNA polymerase (Amaral *et al.* 2007; Zhu *et al.* 2019). In this study, the decrease in sperm motility rate was induced at concentrations where antimycin treatment did not significantly reduce sperm viability. This was due to the decrease in ATP synthesis in the mitochondria by the above mechanism. Thus, oxidative stress from outside the mitochondria rapidly reduces the viability of spermatozoa, whereas oxidative stress inside the mitochondria reduces mobility of the sperm and further reduces sperm survival due to severe stress.

In particular, strong signals of oxidized lipids, which were attenuated by PQQ and vitamin C, were detected in the midpiece region of frozen–thawed sperm. This suggests that excessive oxidative stress, mainly due to mitochondrial dysfunction, significantly reduces the viability of frozen-thawed sperm. It has been reported that abnormal mitochondrial membrane potentials are detected in frozen-thawed spermatozoa and morphological abnormalities in the mitochondria are observed using electron microscopy (Khalil *et al.* 2017). Functional and structural disruption of mitochondria by freezing and thawing increases oxidative stress. On the contrary, ergothioneine improved the viability of frozen-thawed sperm, as well as its positive effect on the decrease in sperm viability due to oxidative stress in the cytoplasm and cell membrane induced by H_2_O_2_ treatment. This indicates that increase in ROS caused by the rapidly increasing temperature and osmotic pressure during the thawing process is a factor that reduces the viability of frozen-thawed sperm. There are many reports of abnormalities in the sperm cell membrane of frozen-thawed spermatozoa in the normality test of the cell membrane using fluorescent staining reagents, which cannot normally pass through the cell membrane (Peña *et al.* 2003; Partyka *et al.* 2010). In other words, in frozen-thawed sperm, oxidative stress on the cell membrane during the thawing process and excessive ROS production due to mitochondrial destruction during incubation can significantly reduce sperm viability.

PQQ has been reported to act directly on the mitochondria by passing through the cell membrane to maintain mitochondrial function (Zhu *et al.* 2006; Rucker *et al.* 2009; Hoque *et al.* 2021). In fact, it has been reported that PQQ not only improves the stability of mitochondrial DNA in boar sperm, but also induces sustained ATP synthesis by improving gene expression from mitochondrial DNA and maintains increased motility for a long time (Zhu *et al.* 2019). Vitamin C is a water-soluble antioxidant that penetrates the cell membrane and exerts ROS-scavenging abilities in both the cytoplasm and mitochondria (Sönmez *et al.* 2005; Hu *et al.* 2010). Similarly, MitoQ and CoQ10 are antioxidant factors that are expected to act on the mitochondria. Although their detailed effects have not been analyzed, their positive effects on sperm have been reported (Zhu *et al.* 2019; Arjun *et al.* 2022). However, ergothioneine, although a water-soluble factor, can exert antioxidant effects not only outside the cell but also inside the cell because it crosses the blood-brain barrier and is taken into the cell via the transporter (OCTN1). OCTN1 is expressed in human spermatozoa, however, its localization has not yet been analyzed (Xuan *et al.* 2003). In this study, ergothioneine did not completely reverse the effect of ROS on the mitochondrial membrane potential induced by antimycin but improved sperm survivability. This suggests that ergothioneine was taken up into the sperm cytoplasm in bull sperm and could not efficiently penetrate the mitochondria, however exerted antioxidant functions in the cytoplasm.

Antioxidants maintain sperm motility, do not degrade sperm quality during handling, and increase sperm motility after freezing and thawing. However, when antioxidant factors are added, it is necessary to consider whether they are taken up by sperm. For example, membrane-impermeable antioxidants such as glutathione have been reported to improve sperm motility, however they are unlikely to have a direct effect (Sławeta and Laskowska 1987; Chatterjee *et al.* 2001; Ansari *et al.* 2012). Another important factor is the stability of the antioxidants. CoQ10 and vitamin C is easily oxidized in solution, decreasing its antioxidant function. Simplicity is also important in reproductive techniques used in animal production, such as artificial insemination, however, antioxidant factors must be mixed immediately before use. Such stability must be verified in the same manner as cell membrane permeability.

Oxidative stress in the testis has a direct negative impact on the spermatogenesis process (Aitken and Roman 2008; Aitken 2020). In addition, it has been reported that oxidative stress in semen increases with age (Vince *et al.* 2018; Baharun *et al.* 2021). In our study using boar sperm, with increasing time of sperm incubation, endogenous ROS was generated in the mitochondria, which reduced sperm motility (Zhu *et al.* 2019). These results indicate not only an artificial increase in exogenous and endogenous oxidative stress, including freezing and thawing, but also a decrease in sperm quality due to increased oxidative stress in vivo. The difference in the action of antioxidant factors revealed in this study is considered to be an important finding for treatment methods that improve spermatogenesis and semen quality deterioration in vivo. In fact, it has been reported that antioxidants improve spermatogenesis and sperm motility in aging males (Agarwal and Sekhon 2010; Leisegang *et al.* 2017; Ioannidou *et al.* 2022).

Thus, to prevent qualitative deterioration of sperm in bulls, it is essential to combine appropriate antioxidant factors according to the oxidative stress that occurs in each sperm compartment. The results obtained from this study provide important insights into the use of antioxidants in semen preparations for artificial insemination.

## Supplementary Information

Below is the link to the electronic supplementary material.

Supplementary file1 Supplemental Figure 1: Gating strategy of flow cytometry. (A) Gating strategy for the selection of single sperm. Using forward scatter (FSC)-A and side scatter (SSC)-A dot plots, cells of similar size and complexity were first selected (R1). In FSC-A and FSC-H dot plots and FSC-A and FSC-W dot plots, similar-size cells were accumulated near the area; thus, using these plots, again similar-size cells were selected (R2, R3). The cells in R3 were used for the below analysis. (B) Histograms of DCFH-DA staining. (C) Histograms of mtSOX deep Red staining. (D) The dot plots of 5,5’,6,6’-tetrachloro-1,1’,3,3’-tetraethylbenzimidazolyl carbocyanine iodide (JC-1) green (x-axis) and red (y-axis). The percentage of JC-1 red-positive sperm (R5) was used for the analysis. (PPTX 349 KB)

## Data Availability

Additional information needed to reanalyze the data reported in this paper is available from the primary contact upon request.
