# Supplementary figures and images for: The protective effects of antioxidants against endogenous and exogenous oxidative stress on bull sperm
Source: In Vitro Cell Dev Biol Anim. 2024 Jul 30;60(9):969–82. doi: 10.1007/s11626-024-00944-w (PMC11534973; doi:10.1007/s11626-024-00944-w)

## Slide 1
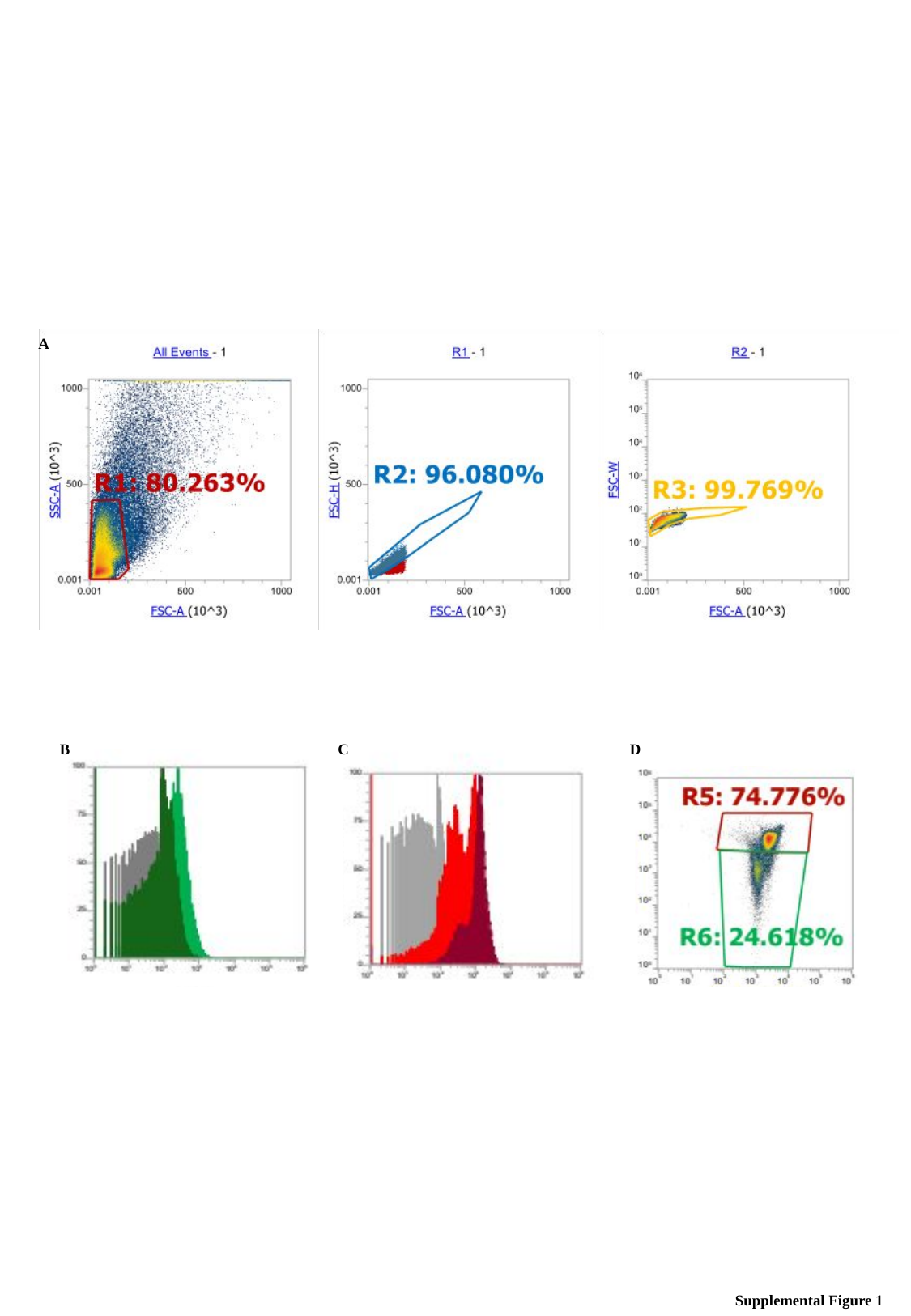

A
B
C
D
Supplemental Figure 1

Supplement: Supplementary file 1 — Supplementary file1 Supplemental Figure 1: Gating strategy of flow cytometry. (A) Gating strategy for the selection of single sperm. Using forward scatter (FSC)-A and side scatter (SSC)-A dot plots, cells of similar size and complexity were first selected (R1). In FSC-A and FSC-H dot plots and FSC-A and FSC-W dot plots, similar-size cells were accumulated near the area; thus, using these plots, again similar-size cells were selected (R2, R3). The cells in R3 were used for the below analysis. (B) Histograms of DCFH-DA staining. (C) Histograms of mtSOX deep Red staining. (D) The dot plots of 5,5’,6,6’-tetrachloro-1,1’,3,3’-tetraethylbenzimidazolyl carbocyanine iodide (JC-1) green (x-axis) and red (y-axis). The percentage of JC-1 red-positive sperm (R5) was used for the analysis. (PPTX 349 KB) [file 11626_2024_944_MOESM1_ESM.pptx]
